# Tobacco use: Health and behavior - R. C. Jiloha

**Published:** 2008

**Authors:** Dr. M. S. Bhatia

**Affiliations:** Department of Psychiatry, UCMS and GTB Hospital, Dilshad Garden, New Delhi - 110 095, India

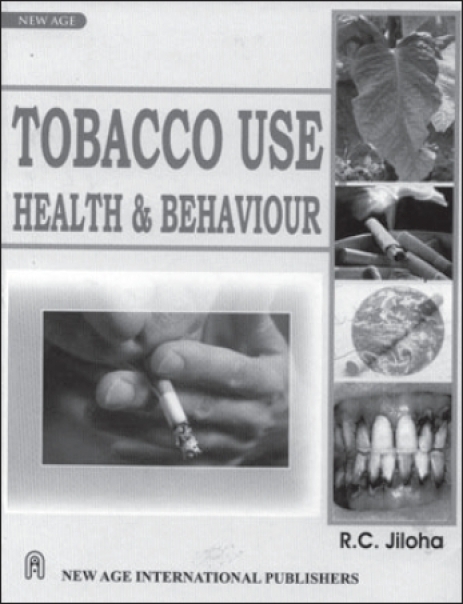



Publisher: New Age International (P) Limited, New Delhi

Price: Rs. 395

Tobacco use has repeatedly been cited by medical authorities as the largest remaining, single, preventable cause of death and disease. However, very little information is available at one place about why it is that people use tobacco, its adverse effects, treatment facilities and control at social and governmental level. The book *Tobacco Use: Health and Behavior*, spread in 12 chapters, covers a wide range of issues related to tobacco. Tobacco is a plant product. Of the 4,000 plants yielding psychoactive substances, about 60 drugs have been in constant use, somewhere in the world, throughout history - with cannabis, opium, coca, tea, coffee, tobacco and alcohol predominantly. Of these, tobacco is one of the recent introductions to the list of psychoactive drugs used in the modern world as smoking and smokeless. The first chapter discusses the history of tobacco use. This chapter provides a critical test for our concepts of reality and the assumptions upon which our present attitudes and policies towards tobacco use are based. This chapter discusses how tobacco assumed political, economic and medical importance since its introduction into the ‘old world’ some 500 years ago. Just within a span of 150 years of its discovery by Spanish navigators, its use spread all over the world. The second chapter describes the magnitude of tobacco use and its global trend. After recognizing the harmful effects of the drug, the Western world has cut down its use; however, there has been a sharp increase in tobacco use in lower- and middle-income countries in the recent years (especially among men). Tobacco use continues to rise in the developing countries. The author describes how the sophisticated, wealthy and powerful drug cartel based in the Western world is dictating its availability in the developing countries. Chapter 3 describes cultivation, curing and commerce of the plant, a most loved and hated member of plant kingdom. Chapters 4 and 5 deal with tobacco's effects and its addiction potential. Nicotine, a pharmacologically active ingredient in tobacco, is highly addictive and responsible for a number of pathophysiological changes in the body. Tobacco smoking is probably the most addictive and dependence-producing, object-specific self-administered gratification known to man, which the author has tried to emphasize. Chapter 6 discusses the vulnerability to tobacco use and how one initiates tobacco use. Smoke exhaled by tobacco smokers pollutes the environment and affects the people who live with the smokers. Women and children are the worst sufferers. This is discussed in chapter 7. Chapters 8 and 9 deal with harmful effects of tobacco on physical and mental health of the user. Smoking harms almost every organ of the body, causes several diseases and reduces the health of the smoker, in general. Chapter 10 describes the treatment for tobacco-cessation. Worldwide, 1.3 billion people smoke and, unless urgent action is taken, 650 million of them will die prematurely due to tobacco use. Current statistics indicate that it will not be possible to reduce tobacco-related deaths over the next 30-50 years, unless adult smokers are encouraged to quit. Chapter 11 discusses tobacco-control. The increasing awareness that Environmental Tobacco Smoke (ETS) is harmful to health places an onus on governments to safeguard public health by providing legislation to protect general public from passive (involuntary) smoking. What will be the future of tobacco use is the subject matter of chapter 12. There will be one billion deaths from tobacco use in the 21^st^ century unless strong and sustained action is taken now. The book provides an account of the current status of knowledge in the field of tobacco use.

This book will be valuable to those wishing to understand tobacco use and to those determining or implementing strategies to control the harm caused by tobacco smoking. This book is meant for post-graduate students in psychiatry, general medicine, public health, psychology and to those undertaking research in the field of addiction, particularly tobacco addiction.

